# Astragaloside IV Relieves Atherosclerosis and Hepatic Steatosis *via* MAPK/NF-κB Signaling Pathway in LDLR^−/−^ Mice

**DOI:** 10.3389/fphar.2022.828161

**Published:** 2022-02-21

**Authors:** Yifan Zhang, Min Du, Jiarou Wang, Ping Liu

**Affiliations:** Department of Cardiology, Longhua Hospital, Shanghai University of Traditional Chinese Medicine, Shanghai, China

**Keywords:** Astragaloside IV, atherosclerosis, hepatic steatosis, inflammation, MAPK/NF-κB signaling pathway

## Abstract

Astragaloside IV (AS-IV) is the main active compound of *Astragalus* membranaceus. In this study, we investigated whether AS-IV could attenuate atherosclerosis and hepatic steatosis in LDLR−/−mice and its potential mechanisms. After 12 weeks of high fat diet, the LDLR−/−mice were randomly divided into four groups. Then, the mice were administrated with 0.9% saline or AS-IV (10 mg/kg) or atorvastatin (1.3 mg/kg) for 12 weeks. Serum lipid profiles and inflammatory cytokines were detected by ELISA, hepatic TC and TG by colorimetric enzymatic kits, gene expression by RT-qPCR, plaque sizes by H&E staining, Oil Red O, liver pathology by H&E staining, collagen content by Masson, α-SMA, caspase-3 and NF-κB p65 production by immunofluorescence staining. MAPK/NF-κB pathway and inflammation related proteins were detected by Western Blot. The results showed that AS-IV decreased the levels of serum lipids, reduced plaque area and increased plaque stability in HFD-induced LDLR^−/−^ mice. AS-IV also decreased the levels of inflammatory cytokines in the serum, aortas and liver tissue, and NF-κB p65 in aortic roots. The phosphorylation of JNK, ERK1/2, p38 and NF-κB, and inflammatory proteins (iNOS, VCAM-1and IL-6) was inhibited in AS-IV-treated group. In summary, AS-IV inhibited inflammation to attenuate atherosclerosis and hepatic steatosis *via* MAPK/NF-κB signaling pathway in LDLR^−/−^ mice.

## Introduction

Atherosclerosis (AS) is the pathological basis of a variety of cardiovascular diseases (CVDs) (Roth et al., 2017). Risk factors include hypertension, diabetes, smoking and adiposity (Herrington et al., 2016). As a lipid-driven inflammatory disease within the arterial wall, AS is always treated with lipid-lowering and anti-inflammatory therapies (Taleb, 2016). In recent decades, statins are used to treat AS, but with lots of adverse effects such as myopathy, rhabdomyolysis, and acute renal failure (Šimić and Reiner, 2015). It is necessary to find more effective methods to prevent and treat AS.

Dyslipidemia, inflammation, and oxidative stress closely link AS with nonalcoholic fatty liver disease (NAFLD). NAFLD has three subtypes: nonalcoholic fatty liver (NAFL), nonalcoholic steatohepatitis (NASH), and related liver cirrhosis. The prevalence of NAFLD has been estimated to be 20–30% in the general population, and associated with several cardiovascular risk factors (Targher et al., 2008). A study has found that the prevalence of CVD is higher in patients with NAFLD (Targher et al., 2010a). NAFLD patients were found to have higher levels of subclinical AS indexes, such as carotidintima-media thickness, and brachial artery flow-mediated dilatation (Villanova et al., 2005; Targher et al., 2006). However, whether NAFLD is the cause or consequence of CVD remains controversial. More researches are needed to explain the role of NAFLD in atherosclerotic cardiovascular events.

**FIGURE 1:**
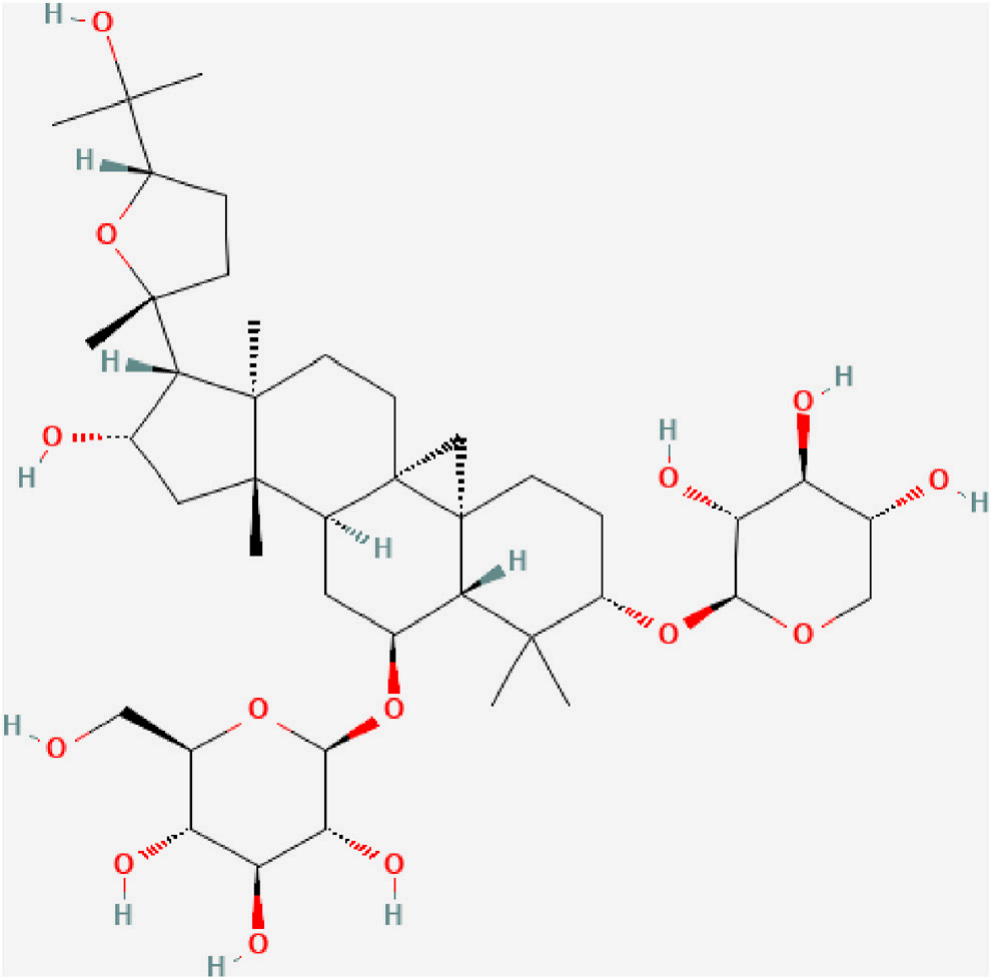
The chemical structure of Astragaloside IV.

Astragaloside IV (AS-IV, C_41_H_68_O_14_, Figure 1), a cycloartane-type triterpene glycoside chemical, is a major compound in the aqueous extract from *Astragalus* membranaceus (Zhang et al., 2020). Many studies have demonstrated that AS-IV can protect the cardiovascular system, lungs, kidneys and brain, an effect attributed to its anti-inflammatory, antioxidant, anti-apoptotic and immunoregulation properties (Yan et al., 2017; Meiqian et al., 2018; Zhang et al., 2019; Tan et al., 2020). It has been shown that AS-IV could counter inflammation through multiple signaling pathways, such as Toll like receptor four/nuclear factor–kappa B (TLR4/NF-κB) signaling pathway, mitogen-activated protein kinases (MAPK) signaling pathway, and Janus kinase two/signal transducer and activator of transcription 6 (JAK2/STAT6) signaling pathway (Leng et al., 2019; Yang and Wang, 2019; Hsieh et al., 2020).

In this study, we investigated whether AS-IV can attenuate HFD-induced AS and hepatic steatosis by repressing MAPK/NF-κB pathway in the aortas and liver tissue of LDLR^−/−^ mice.

## Methods

### Materials and Reagents

Astragaloside IV was extracted from PP (purity: 99.63%, available at:http://www.cdmust.com) by Mansite Biological Company (Chengdu, China). The high-fat diet (HFD, 78.85% normal diet+21% lard +0.15% cholesterol) was bought from SYSE Biotechnology Co., Ltd. (Changzhou, China); atorvastatin calcium tablets from Pharmacy of Longhua Hospital Affiliated to Shanghai University of Traditional Chinese Medicine; enzyme immunosorbent assay (ELISA) kits for mouse IL-1β, IL- 6 and TNF-α from Shanghai XiTang Biological Technology Co., Ltd. (Shanghai, China); oil red O staining kit, Masson staining kit, total cholesterol assay kit and triglyceride assay kit from Jiancheng Bioengineering Institute of Nanjing (Nanjing, China); primary antibodies against ERK, p-ERK, P38 MAPK, p-P38 MAPK, JNK, p-JNK, NF-κB, p-NF-κB, IL-6, iNOS, VCAM-1, α-SMA, caspase 3 and GAPDH from Cell Signaling Technology Inc. (Beverly, Massachusetts, United states). ECL Star kit and Hematoxylin, Eosin (H&E) Staining Kit, 4′, 6-diamindino-2-phenylindole (DAPI), Triton X-100, radio immunoprecipitation assay (RIPA), and BCA protein assay kit were from Beyotime Biotechnology (Shanghai, China); primers for IL-1β, IL-6 and TNF-α from Sangon Biotech Co., Ltd. (Shanghai, China); primeScript RT Reagent Kit for RT-PCR amplification and TB Green Premix EX Taq from TAKARA Biomedical Technology Co., Ltd. (Beijing, China); RNA Purification Kit from EZBioscience (Shanghai, China).

### Animals and Treatment

Six-week-old LDLR^−/−^ male mice (18–22 g) were obtained from the GemPharmatech Co., Ltd. (Nanjing, Jiangsu, http://www.gempharmatech.com) (SCXK 2018-0008). All mice were housed in the Animal Center of Longhua Hospital Affiliated to Shanghai University of Traditional Chinese Medicine. After 1 week of environmental adaptation, all mice were randomly assigned to four groups: control group (CON), model group (MOD), Astragaloside IV group (AS- IV, 10 mg/kg), and Atorvastatin group (ATO, 1.3 mg/kg). The mice in CON group were fed with normal diet for 24 weeks, and those in other groups with HDF. The dose of AS-IV is based on the references (Min Li et al., 2017; Liu et al., 2018; Yang et al., 2020). The dose of ATO is calculated according to the human and mouse dose conversion, and refers to the reference (Xu et al., 2017). After the mice were fed for 12 weeks, the LDLR^−/−^ mice in AS- IV group were intraperitoneally injected with AS-IV, and ATO group received oral atorvastatin for 12 weeks. CON and MOD groups were injected with the same volume of 0.9% saline. After a 12-h fasting, all the mice were sacrificed via carbon dioxide asphyxiation. The animal study was approved by the Ethics Committee of Longhua Hospital Affiliated to Shanghai University of Traditional Chinese Medicine (No. 2019-N002, shown in Supplementary Material).

### Serum Biochemical Analysis and Determination of Hepatic Lipids

Alanine aminotransferase (ALT), aspartate aminotransferase (AST), total cholesterol (TC), and triglyceride (TG) levels in the blood serum were analyzed in the Clinical Laboratory Department of Longhua Hospital Affiliated to Shanghai University of Traditional Chinese Medicine. The hepatic levels of TC and TG were measured using colorimetric enzymatic kits.

### Determination of Cytokine

The concentrations of IL-1β, IL-6 and TNF-α in the serum were detected by ELISA kits according to the manufacturer’s instructions. The final concentrations were calculated in accordance with corresponding standard curves.

### Histopathological Analysis

The aortic roots and liver tissue were fixed in 4% paraformaldehyde for 48 h. The aortic roots were embedded in the optimal cutting temperature (OCT) compound and cut into circular sections (10 μm) with a microtome. The sections of aortic root were stained with H&E, Oil Red O and Masson solution. The lipid-rich plaque areas were observed under the microscope and analyzed by ImageJ software. Liver tissues that had been fixed in 4% paraformaldehyde were dehydrated in ethanol and finally embedded in paraffin. Paraffin sections were then sliced 10 µm thick and stained with H&E.

### Western Blotting

The thoracic aorta or liver tissue were lysed in RIPA lysis solution for 30 min. The supernatant was collected after a 10-min centrifugation. The total protein was quantified using BCA kit, separated on 10% SDS-PAGE and transferred onto the PVDF membranes. The PVDF membranes were immersed in 5% BSA in Tris-buffered saline with Tween-20 (TBST) for 1 h. The blocked PVDF membranes were incubated with primary antibodies (ERK, p-ERK, P38 MAPK, p-P38 MAPK, JNK, p-JNK, NF-κB, p-NF-κB, IL-6, iNOS, VCAM-1and GAPDH) diluted with 5% BSA in TBST at 4°C overnight. Having been washed with TBST, the membranes were immersed in secondary antibodies for 1 h. The contents of proteins were imaged using ECL reagent and photographed using the ChemiScope 6,000. Finally, the density of protein bands was analyzed with ImageJ software.

### Real-Time Quantitative PCR (RT-qPCR) Assay

Total RNA was extracted for the mice aorta tissue and liver tissue using RNA Purification Kit, and reverse-transcribed into cDNA using PrimeScript RT Reagent Kit following the standard protocol. The Real-Time Quantitative PCR (RT-qPCR) assay was conducted using TB Green PremixEX Taq with the Applied Biosystems 7,500 Real-Time PCR System. The amplification parameters were set at 95°C for 1 min, followed by 40 cycles of 95°C for 5 s and 58°C for 15 s, 72°C for 30 s, and 95°C for 15 s. The relative expression of mRNA was normalized to that of β-actin. All primer sequences used are listed in Table 1.

**TABLE 1:** List of primers for real-time qPCR analysis.

Gene		Oligonucleotide sequence
β-Actin	Forward	5′- GAG​ACC​TTC​AAC​ACC​CCA​GC - 3′
—	Reverse	5′- ATG​TCA​CGC​ACG​ATT​TCC​C- 3′
IL-1β	Forward	5′- GCT​TCA​GGC​AGG​CAG​TAT​CA- 3′
—	Reverse	5′- TGC​AGT​TGC​TAA​TGG​GAA​CG- 3′
IL-6	Forward	5′-AAA​GCA​GCA​AAG​AGG​CAC​TG- 3′
—	Reverse	5′-TAC​CTC​AAA​CTC​CAA​AAG​ACC​AG- 3′
TNF-α	Forward	5′- CCC​TCC​AGA​AAA​GAC​ACC​ATG- 3′
—	Reverse	5′- CAC​CCC​GAA​GTT​CAG​TAG​ACA​G- 3′

### Immunofluorescence Study

Frozen slices of aortic sinus were used for immunofluorescence study. Having been fixed with 4% paraformaldehyde for 15 min and permeabilized by 0.1% Triton X-100 for 10 min, the cells were blocked with PBS containing 5% BSA for 1 h. Then, the frozen slices were incubated with anti-α-SMA or caspase 3 or anti-NF-κB p65 at 4°C overnight. On the following day, the cells were washed with PBS and incubated with goat anti-rabbit IgG H&L for 1 h. Finally, the cells were treated with DAPI. All images were captured with a fluorescence microscope.

### Statistical Analysis

All experimental results were shown as the mean ± standard deviation (SD). The statistical analyses were accomplished with GraphPad Prism 7.0. software and SPSS 21.0. Significant differences between groups were defined by the one-way ANOVA. *p* < 0.05 was considered statistically significant.

## Results

### Effect of Astragaloside IV on Serum Lipid Profiles and Atherosclerotic Lesions in the Aortic Sinus of Mice

**FIGURE 2:**
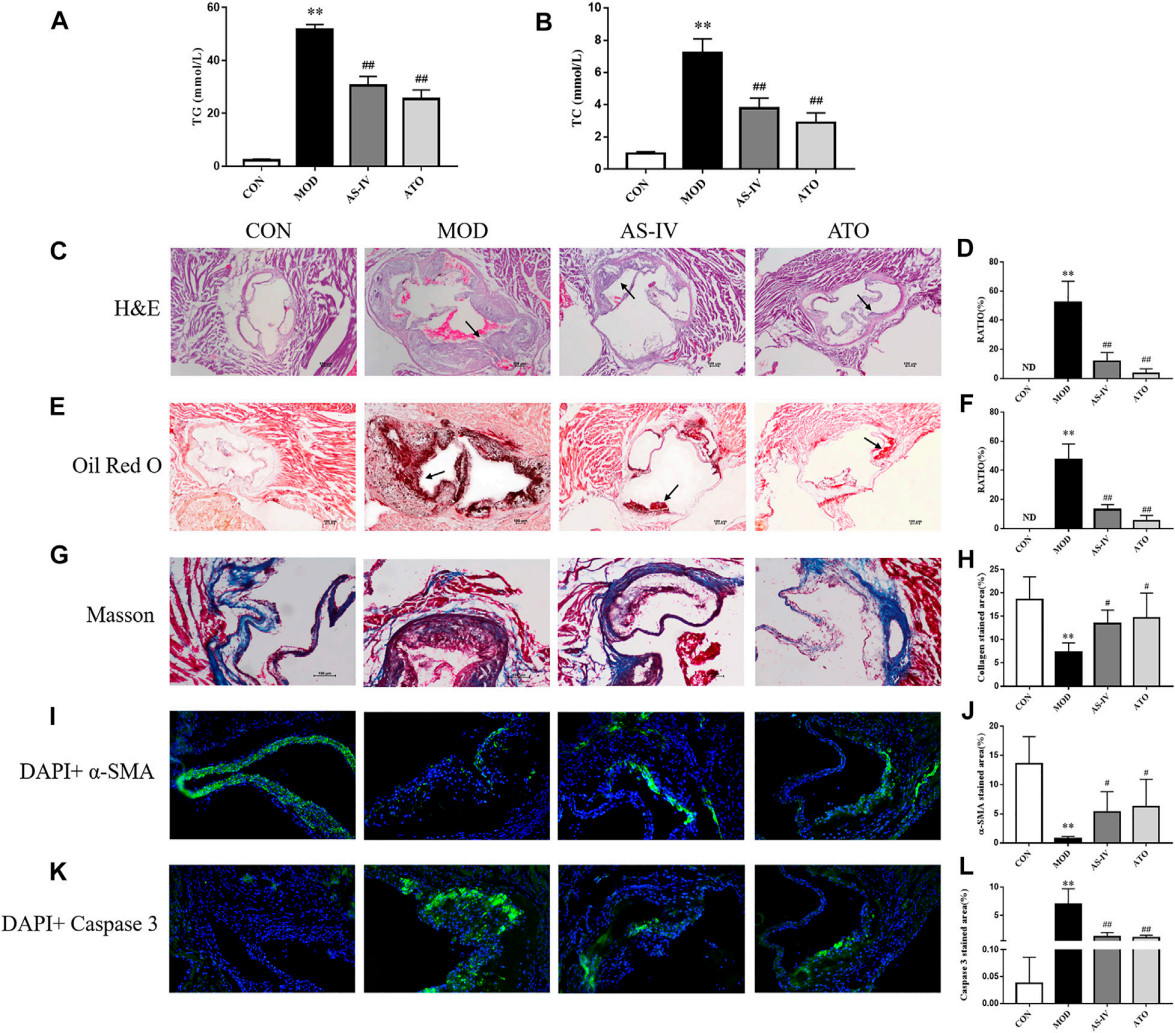
Serum lipid profiles and atherosclerosis of aortic sinus in the LDLR^−/−^ mice. **(A–B)** TC and TG levels of serum were detected, *n* = 8. H&E **(C)** and Oil Red O staining **(E)** were used to detect the progress of lipid-rich plaques, magnification ×40, *n* = 6. **(B,D)** The areas of lesion were calculated respectively by ImageJ analysis software. Masson staining **(G)** was used to detect the collagen content followed by the quantification **(H)**, magnification ×100, *n* = 6. **(I–L)** Representative photomicrographs of aortic root sections stained with α-SMA **(I)**, and caspase 3 **(K)** in atherosclerotic plaque followed by the quantification **(J,L)**, magnification ×200, *n* = 6. CON means control group, MOD means model group, AS-IV means Astragaloside IV group, ATO means Atorvastatin group. TC, total cholesterol; TG, triglyceride; ND, not detected; RATIO = plaque area/aortic sinus lumen area ×100%. Data are expressed as mean ± SD. **p < 0.01 versus CON group; #*p* < 0.05, ##*p* < 0.01 versus MOD group.

To explore the effect of AS-IV on blood lipids in AS mice, the serum levels of TC and TG were detected. As shown in Figures 2A,B, TC and TG serum levels were lower in CON group. The TC and TG serum levels were significantly increased in MOD group (*p* < 0.01); while after 12 weeks’ intervention with AS-IV or ATO, the indexes were lower than those in MOD group (*p* < 0.01). The above results showed that AS-IV could correct lipid metabolism in AS mice.

Lipid plaques in the aorta were observed by H&E and Oil Red O staining. As shown in Figures 2C–F, the lipid accumulation in the aortic roots was more significant in MOD group, but not detected in CON group (*p* < 0.01). In contrast, the atherosclerotic lesion reduced in AS-IV-treated and ATO-treated groups (*p* < 0.01), compared to MOD group. The above results suggested that AS-IV could reduce AS plaques in HFD-induced-LDLR−/− mice.

The characteristics of a vulnerable plaque include a high necrotic core size and a low fibrous cap area. Then the collagen content and the expression levels of α-SMA and caspase 3 were detected by immunofluorescence to explore the effect of AS-IV on the stability of atherosclerotic plaques. Figures 2G–L showed that the atherosclerotic lesions of the MOD group had a lower collagen content, a lower expression level of α-SMA and a higher expression level of caspase 3 than CON group (*p* < 0.01). AS-IV or ATO increased collagen content and α-SMA expression, and decreased caspase 3 expression (*p* < 0.05). The results suggested that the stability of atherosclerotic plaque could be enhanced by AS-IV, reducing the risk of plaque rupture.

### Effect of Astragaloside IV on Transaminase, Liver Lipogenesis and Hepatic Steatosis in Mice

**FIGURE 3:**
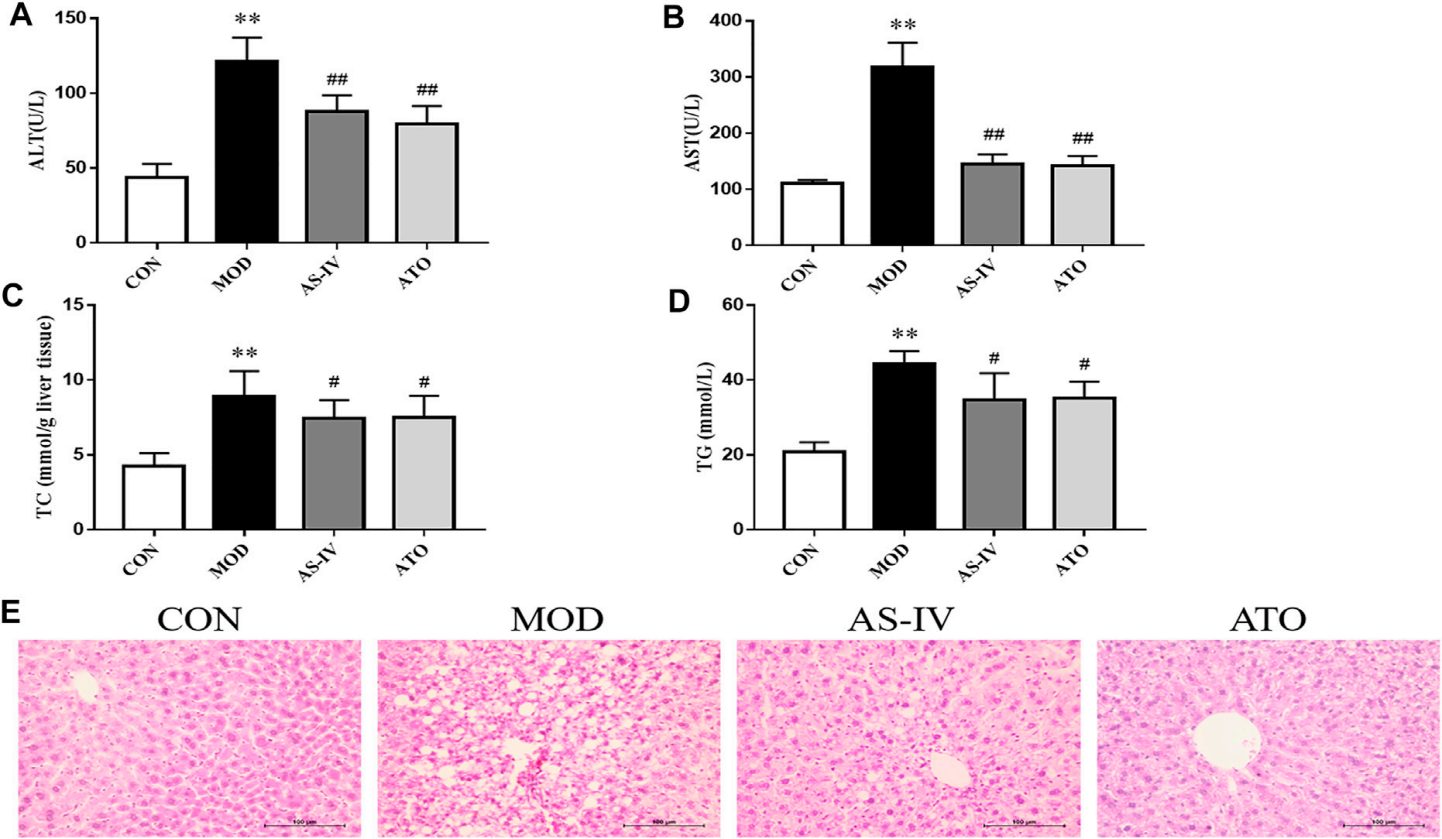
Transaminase, liver lipogenesis and hepatic steatosis in the LDLR^−/−^ mice. **(A,B)** ALT and AST levels of serum were detected, *n* = 8. **(C,D)** Hepatic TC and TG levels were detected by colorimetric enzymatic kits, *n* = 8. **(E)** Representative histological change of steatosis in liver sections stained with H&E, magnification ×400. CON means control group, MOD means model group, AS-IV means Astragaloside IV group, ATO means Atorvastatin group. ALT, alanine aminotransferase; AST, aspartate aminotransferase; TC, total cholesterol; TG, triglyceride. Data are expressed as mean ± SD. **p < 0.01 versus CON group; ##*p* < 0.01 versus MOD group.

Long-term HFD leads to lipid deposition in the liver, leading to liver inflammation. To investigate the effect of AS-IV, we also tested serum ATL and AST levels in each group. As shown in Figures 3A,B, the serum AST and ALT levels in the MOD group were significantly higher than those in the CON group (*p* < 0.01). AS-IV and ATO treatments showed a significantly decreased level of serum AST and ALT (*p* < 0.01). This suggested that AS-IV significantly reduced the release of transaminase.

To investigate the effect of AS-IV on HFD-induced hepatic steatosis, we observed H&E staining of liver tissue, and examined the hepatic triglyceride and cholesterol accumulation (Figures 3C–E). After 12 weeks of HFD, the MOD group mice showed significantly liver fat deposition. 12-week administration of AS-IV and ATO reduced the liver lipid deposition through microscope observation. Hepatic TC and TG levels in the MOD group were significantly higher than those in the CON group (*p* < 0.01). AS-IV and ATO significantly reduced the hepatic TC and TG content (*p* < 0.01), indicating that hepatic steatosis was attenuated by AS-IV.

### Effect of Astragaloside IV on Inflammation Cytokines in Mice

**FIGURE 4:**
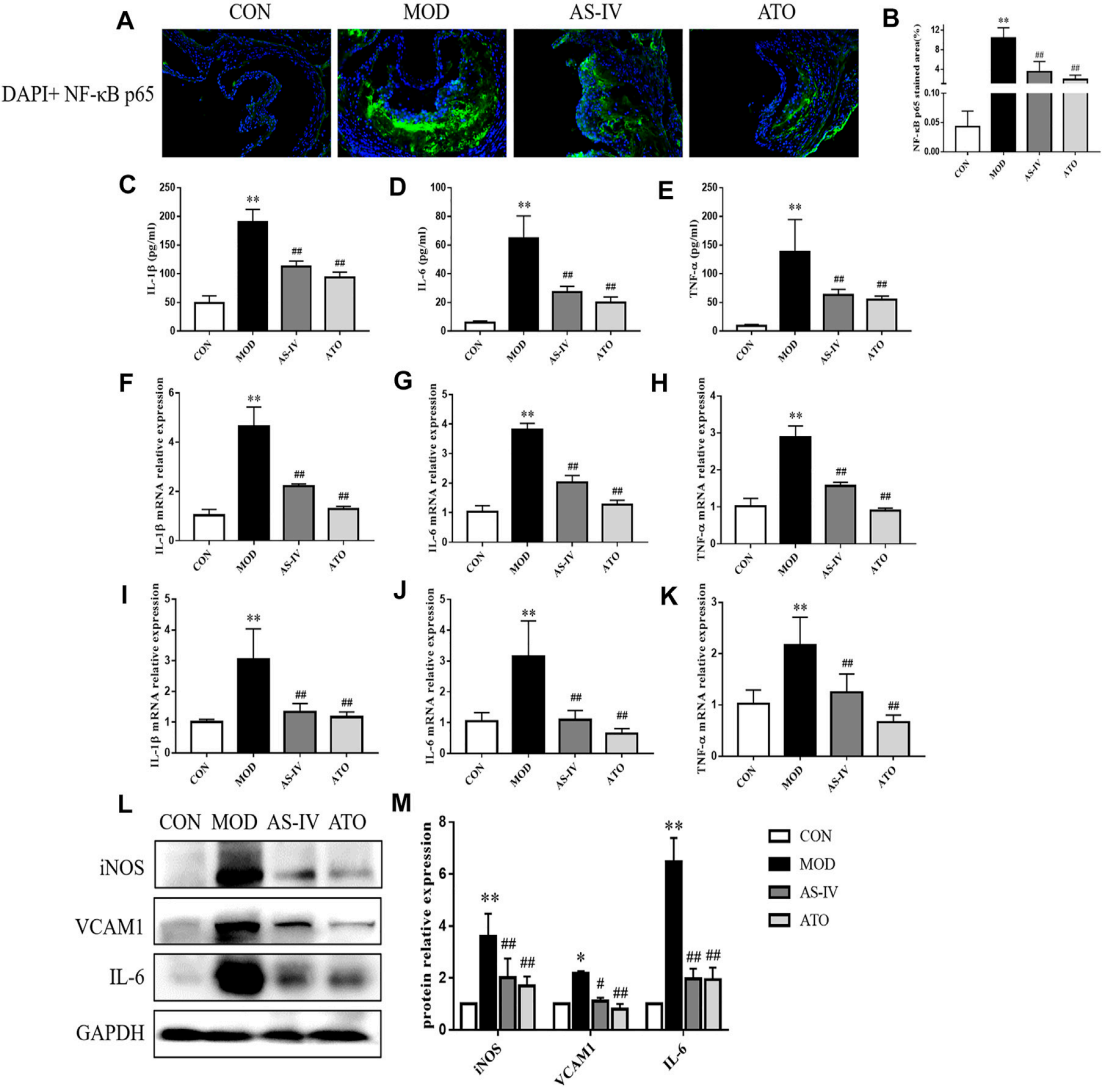
Inflammatory cytokines in the LDLR^−/−^ mice. **(A,B)** Representative photomicrographs of aortic root sections stained with NF-κB P65 in atherosclerotic plaque followed by the quantification, magnification ×200, *n* = 6. **(C–E)** Production of IL-1β, IL-6 and TNF-α in serum were measured by ELISA kit, *n* = 8. Aorta **(F–H)** and liver tissue **(I–K)** gene expression of IL-1β, IL-6 and TNF-α were detected by RT-qPCR method, *n* = 8. **(L)** Protein was extracted from mice aorta. Then the levels of iNOS, VCAM-1 and IL-6 were determined by western blotting assay, *n* = 3. **(M)** The quantitative results were depicted. CON means control group, MOD means model group, AS-IV means Astragaloside IV group, ATO means Atorvastatin group. Data are expressed as mean ± SD. **p < 0.01 versus CON group; ##*p* < 0.01 versus MOD group.

In order to explore the effect of AS-IV on the inflammation, immunofluorescence staining of NF-κB p65 was performed and the expression levels of inflammation cytokines were detected. As shown in Figures 4A, B, NF-κB p65 content was significantly increased in the atherosclerotic lesions of the MOD group (*p* < 0.01). AS-IV and ATO inhibited the increase of NF-κB p65 (*p* < 0.05).

Then, the serum levels of inflammatory cytokines IL-1β, IL-6, and TNF-α were detected by ELISA kits (Figures 4C–E). The levels of IL-1β, IL-6 and TNF-α in the MOD group increased compared with those in the CON group (*p* < 0.01). While 12 weeks’ intervention with AS-IV or ATO significantly decreased the levels of IL-1β, IL-6 and TNF-α expression (*p* < 0.01). The mRNA levels of those inflammatory cytokines in mice aortas and liver tissue were detected by RT-qPCR (Figures 4F–K). Similarly, AS-IV or ATO decreased the mRNA levels of IL-1β, IL-6 and TNF-α expression in mouse aortas and liver tissue (*p* < 0.01). The expression levels of inflammatory proteins iNOS, VCAM-1, and IL-6 were detected by Western blotting (Figures 4L,M). AS-IV also down-regulated the expression of iNOS, VCAM-1, and IL-6 (*p* < 0.01). The above results suggested that AS-IV could reduce the inflammatory response through inhibiting the generation of inflammatory factors.

### Effect of Astragaloside IV on MAPK Signal Pathway in Mice

**FIGURE 5:**
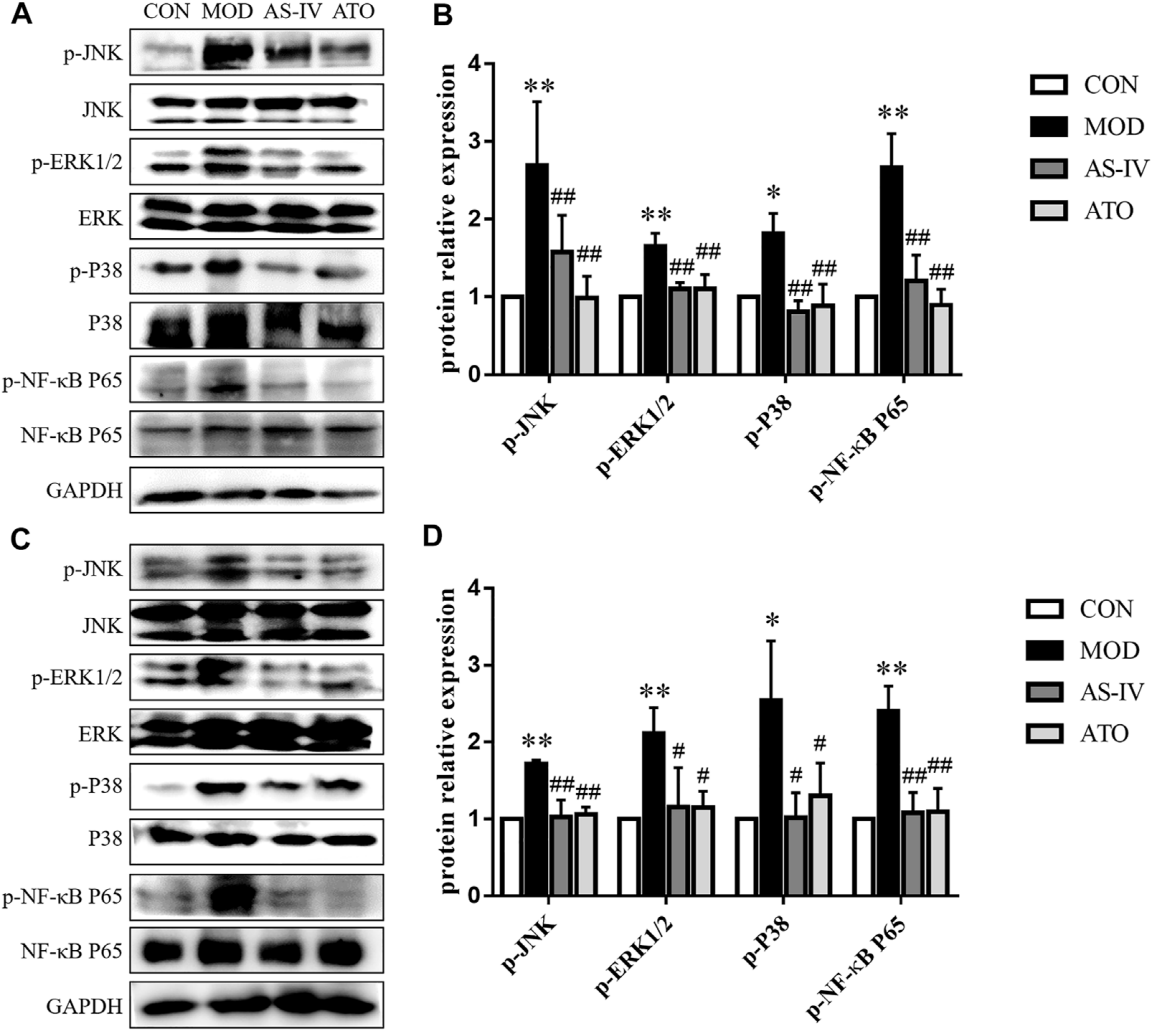
MAPKs signaling pathway in the LDLR^−/−^ mice. Protein was extracted from mice aorta **(A)** and liver tissue **(C)**. Then the total and phosphorylated levels of JNK, ERK1/2, p38 and NF-κB P65 were determined by western blotting assay, *n* = 3. **(B,D)** The quantitative results were depicted. CON means control group, MOD means model group, AS-IV means Astragaloside IV group, ATO means Atorvastatin group. Data are expressed as mean ± SD. **p < 0.01 versus CON group; ##*p* < 0.01 versus MOD group.

Long-term fat deposition could lead to inflammation. Inflammation plays an important role in the development of AS and NAFLD. MAPK and NF-κB are critical factors in inflammatory responses. To determine whether the beneficial effect of AS-IV on the aortic roots and liver was associated with MAPK/NF-κB signaling pathway, we examined the phosphorylation of JNK, ERK1/2, p38 and NF-κB p65 in the aortas and liver tissue (Figures 5A–D). Compared to the CON group, the Western blotting showed that the protein expression of MAPK/NF-κB in the MOD group was markedly upregulated and restored after AS-IV administration (*p* < 0.01). These results suggested AS-IV could mitigate the activation of MAPK/NF-κB in atherosclerotic mice.

## Discussion

The development of AS requires lipid accumulation and inflammatory response (Moore et al., 2013). AS-IV is a type of polysaccharide extracted from *Astragalus* membranaceus, which is a traditional Chinese herbal medicine for CVD (Zhang et al., 2011; Ma et al., 2013). In this study, we investigated whether AS-IV could alleviate AS and hepatic steatosis, and uncovered the underlying mechanism. The results showed that AS-IV inhibited the development of AS and hepatic steatosis, which may be attributed to its beneficial effects on lipid metabolism and inflammation. Mechanistically, we found that AS-IV fights against inflammation by inactivating MAPK/NF-κB signaling pathway. Although our results show that atorvastatin has better lipid metabolism and anti-inflammatory effects. And studies have confirmed that statins affect atherosclerosis through several other effects, such as plaque stabilization, reducing inflammation, reversing endothelial dysfunction, and reducing thrombosis (Girotra et al., 2012; Okuyama et al., 2015; Niedzielski et al., 2020). Myopathy, rhabdomyolysis, acute renal failure and liver enzyme abnormalities were the most frequently reported adverse events of statins (Šimić and Reiner, 2015; Newman et al., 2019). Therefore, we need to find more effective traditional Chinese medicine for the treatment of atherosclerosis. AS-IV has no obvious toxicity or adverse reactions (Yu et al., 2007). It has been documented that oral administration of AS-IV did not affect liver and renal function (Gui et al., 2012). The numerous experiments provide substantial evidence for AS-IV prevented the progression of cardiovascular diseases (Qin et al., 2015; Xiong-Zhi Li et al., 2017; Nanding Wang et al., 2020). Besides, recent studies have shown the potential therapeutic benefits that AS-IV confers in the treatment of kidney injury and liver cirrhosis (Xu et al., 2014; Xin et al., 2015; Wei et al., 2019).

Increased plasma cholesterol and triglyceride levels are risk factors for AS (Beverly and Budoff, 2020). Chylomicrons, VLDL and LDL carry these lipids into the arterial wall where the lipids accumulate and deposit to initiate early-stage AS (Ference et al., 2018). LDL is prone to undergoing various atherogenic modifications, the most common of which is oxidation (Quinn et al., 1987). Modified LDL serves as the primary source of cholesterol accumulation and chronic inflammation in atherosclerotic plaques (Tabas et al., 2015). Oxidized LDL (ox-LDL) can be taken up by macrophages, thereby turning them into foam cells presented as fat streaks in the early stage of AS (Maguire et al., 2019). Therefore, lipid-lowering is the cornerstone in the treatment of AS (Mourikis et al., 2020). The results of our study showed that AS-IV decreased serum lipid levels and plaque accumulation in the aortic roots. With the development of plaques, their thinning fibrous caps and enlarging necrotic cores make plaques brittle, resulting in severe cardiovascular events (Insull, 2009). Activation of caspase 3 regulates inflammation and cell death. Previous studies have identified cleaved caspase 3 in atherosclerotic plaques, where it colocalizes with dead macrophages and lipid-rich plaque components (Hutter et al., 2004; Kong et al., 2019). It has also been shown in an animal study that caspase-3 inhibitors protect cholesterol-loaded hepatocytes (Gan et al., 2014). We found that AS-IV increased collagen and α-SMA content, and reduced caspase 3 content in the plaque. These results suggested that AS-IV improved AS through correcting lipid metabolism and enhancing plaque stability.

The hepatic functions, mainly determining the cholesterol metabolism and lipid profile in the serum, are critical for AS development (Purnak et al., 2011). The results showed that AS-IV reduced the levels of hepatic TC and TG, and liver fat deposition. A study has demonstrated that elevated ALT levels, as a marker for NAFLD, are associated with increased CVD-related mortality in Koreans (Yun et al., 2009). AS-IV also decreased the levels of ALT and AST. These results indicate that AS-IV reduces hepatic steatosis, a beneficial effect in treating AS.

Lipid metabolism is also associated with inflammation. Accumulated evidence demonstrates that ox-LDL produces pathophysiological effects, including the release of proinflammatory cytokines, overexpression of cell adhesion molecules, and impairment of endothelium-dependent vasorelaxation (Di et al., 2017). Endothelial cells dysfunction, due to the increased ox-LDL, can activate the inducible isoform of nitric oxide synthase (iNOS) (Stancu et al., 2012; Gliozzi et al., 2019). In addition, ox-LDL can activate TLR4/NF-κB signaling pathway via lipoprotein receptor-1 (Bai et al., 2017; Balzan and Lubrano, 2018), thus increasing the levels of iNOS and vascular cell adhesion molecule 1 (VCAM-1) (Zhong et al., 2018; Gliozzi et al., 2019). These molecules enhance inflammation and exacerbate AS. Therefore, anti-inflammatory strategies are essential for the treatment of AS (Pedro-Botet et al., 2020). Our results showed that AS-IV restored the serum levels of IL-1β, IL-6 and TNF-α, and the mRNA expression levels of these cytokines in mice aortas. AS-IV also repressed the increase of inflammatory proteins (iNOS, VCAM-1 and IL-6) induced by HFD. These results indicate that AS-IV attenuates AS through inhibiting the release of inflammatory factors.

There is no doubt that inflammatory responses become more severe in the stage of NASH. Some studies have shown that patients with NASH are more likely to develop AS compared with those with simple steatosis (Targher et al., 2010b; Söderberg et al., 2010). Studies have shown that hepatic inflammation occurs earlier than the formation of early aortic lesions, and partial inflammatory factors that lead to AS are generated in the liver (Purnak et al., 2011). Excessive accumulation of lipids results in hepatic inflammation, activating interferon γ, IL-1, TNF-α and other inflammatory signal pathways to promote AS (Kleemann et al., 2007). Therefore, alleviating hepatic inflammation has potential benefits in AS treatment. In the AS-IV group, the mice showed decreased mRNA expression levels of IL-1β, IL-6 and TNF-α. These results suggest that AS-IV ameliorates hepatic steatosis through suppressing the expression of inflammatory factors.

Both MAPK and NF-κB are key signaling pathways responsible for inflammation in the development of AS and NAFLD (Hopkins, 2013; Hernández-Aquino and Muriel, 2018). Stimulated by endotoxin or lipopeptide, TLR4 triggers the myeloid differentiation factor 88-dependent pathway, rapidly activates the MAPK/NF-κB signaling pathway, and upregulates the transcription of inflammatory factors, all stoking up hepatic inflammation to aggravate AS (Wang et al., 2014; Wu et al., 2018). Recent research found that inhibiting the activation of MAPK signaling pathway could inhibit AS (Yiru Wang et al., 2020; Jiang et al., 2020). And suppressing MAPK/NF-κB signaling pathway could mitigate NASH(Li et al., 2020). Existing studies showed that AS-IV inhibits inflammation and oxidation by inhibiting NF-κB, MAPK signaling pathways (Zhang and Frei, 2015; Hsieh et al., 2020). And the potential effects of AS-IV on cardiac disease have been described by regulating the MAPK signaling pathway (Sun et al., 2021). AS-IV administration could downregulate the phosphorylation of JNK, ERK1/2, p38 and NF-κB p65 in the aortas and liver tissue. These suggest that AS-IV exerts anti-AS and anti-inflammation effects by suppressing HFD-induced activation of MAPK/NF-κB signaling pathway.

In conclusion, AS-IV can attenuate AS development and hepatic steatosis via improving the lipid metabolism and inhibiting inflammation through suppressing MAPK/NF-κB signaling pathway. Therefore, AS-IV may act as a promising anti-atherosclerotic drug. Further *in vitro* studies should be conducted to clarify the beneficial effects of AS-IV on AS and hepatic steatosis. Activation of MAPK may affect apolipoprotein B stability and/or degradation, providing a potential target for AS-IV against atherosclerosis and hepatic steatosis.

## Data Availability

The original contributions presented in the study are included in the article/Supplementary Material, further inquiries can be directed to the corresponding author.

## References

[B1] Bai X. L. Yang X. Y. Li J. Y. Ye-Li L. Jia X. Xiong Z. F. (2017). Cavin-1 Regulates Caveolae-Mediated LDL Transcytosis: Crosstalk in an AMPK/eNOS/NF-κB/Sp1 Loop. Oncotarget 8 (61), 103985–103995. 10.18632/oncotarget.21944 29262615 PMC5732781

[B2] Balzan S. Lubrano V. (2018). LOX-1 Receptor: A Potential Link in Atherosclerosis and Cancer. Life Sci. 198, 79–86. 10.1016/j.lfs.2018.02.024 29462603

[B3] Beverly J. K. Budoff M. J. (2020). Atherosclerosis: Pathophysiology of Insulin Resistance, Hyperglycemia, Hyperlipidemia, and Inflammation. J. Diabetes 12 (2), 102–104. 10.1111/1753-0407.12970 31411812

[B4] Di X. Tang X. Di X. (2017). Montelukast Inhibits Oxidized Low-Density Lipoproteins (Ox-LDL) Induced Vascular Endothelial Attachment: An Implication for the Treatment of Atherosclerosis. Biochem. Biophys. Res. Commun. 486 (1), 58–62. 10.1016/j.bbrc.2017.02.125 28246014

[B5] Ference B. A. Graham I. Tokgozoglu L. Catapano A. L. (2018). Impact of Lipids on Cardiovascular Health: JACC Health Promotion Series. J. Am. Coll. Cardiol. 72 (10), 1141–1156. 10.1016/j.jacc.2018.06.046 30165986

[B6] Gan L. T. Van Rooyen D. M. Koina M. E. McCuskey R. S. Teoh N. C. Farrell G. C. (2014). Hepatocyte Free Cholesterol Lipotoxicity Results from JNK1-Mediated Mitochondrial Injury and Is HMGB1 and TLR4-dependent. J. Hepatol. 61 (6), 1376–1384. 10.1016/j.jhep.2014.07.024 25064435

[B7] Girotra S. Murarka S. Migrino R. Q. (2012). Plaque Regression and Improved Clinical Outcomes Following Statin Treatment in Atherosclerosis. Panminerva Med. 54 (2), 71–81. 22525562

[B8] Gliozzi M. Scicchitano M. Bosco F. Musolino V. Carresi C. Scarano F. (2019). Modulation of Nitric Oxide Synthases by Oxidized LDLs: Role in Vascular Inflammation and Atherosclerosis Development. Int. J. Mol. Sci. 20 (13), 3294. 10.3390/ijms20133294 PMC6651385 31277498

[B9] Gui D. Guo Y. Wang F. Liu W. Chen J. Chen Y. (2012). Astragaloside IV, a Novel Antioxidant, Prevents Glucose-Induced Podocyte Apoptosis *In Vitro* and *In Vivo* . PloS one 7 (6), e39824. 10.1371/journal.pone.0039824 22745830 PMC3382154

[B10] Hernández-Aquino E. Muriel P. (2018). Beneficial Effects of Naringenin in Liver Diseases: Molecular Mechanisms. World J. Gastroenterol. 24 (16), 1679–1707. 10.3748/wjg.v24.i16.1679 29713125 PMC5922990

[B11] Herrington W. Lacey B. Sherliker P. Armitage J. Lewington S. (2016). Epidemiology of Atherosclerosis and the Potential to Reduce the Global Burden of Atherothrombotic Disease. Circ. Res. 118 (4), 535–546. 10.1161/circresaha.115.307611 26892956

[B12] Hopkins P. N. (2013). Molecular Biology of Atherosclerosis. Physiol. Rev. 93 (3), 1317–1542. 10.1152/physrev.00004.2012 23899566

[B13] Hsieh H. L. Liu S. H. Chen Y. L. Huang C. Y. Wu S. J. (2020). Astragaloside IV Suppresses Inflammatory Response via Suppression of NF-Κb, and MAPK Signalling in Human Bronchial Epithelial Cells. Arch. Physiol. Biochem., 1–10. 10.1080/13813455.2020.1727525 32057253

[B14] Hutter R. Valdiviezo C. Sauter B. V. Savontaus M. Chereshnev I. Carrick F. E. (2004). Caspase-3 and Tissue Factor Expression in Lipid-Rich Plaque Macrophages: Evidence for Apoptosis as Link between Inflammation and Atherothrombosis. Circulation 109 (16), 2001–2008. 10.1161/01.CIR.0000125526.91945.AE 15078795

[B15] Insull W. Jr. (2009). The Pathology of Atherosclerosis: Plaque Development and Plaque Responses to Medical Treatment. Am. J. Med. 122 (1 Suppl. l), S3–s14. 10.1016/j.amjmed.2008.10.013 19110086

[B16] Jiang L. Qiao Y. Wang Z. Ma X. Wang H. Li J. (2020). Inhibition of microRNA-103 Attenuates Inflammation and Endoplasmic Reticulum Stress in Atherosclerosis through Disrupting the PTEN-Mediated MAPK Signaling. J. Cel Physiol 235 (1), 380–393. 10.1002/jcp.28979 31232476

[B17] Kleemann R. Verschuren L. van Erk M. J. Nikolsky Y. Cnubben N. H. Verheij E. R. (2007). Atherosclerosis and Liver Inflammation Induced by Increased Dietary Cholesterol Intake: a Combined Transcriptomics and Metabolomics Analysis. Genome Biol. 8 (9), R200. 10.1186/gb-2007-8-9-r200 17892536 PMC2375038

[B18] Kong Y. Y. Li G. Q. Zhang W. J. Hua X. Zhou C. C. Xu T. Y. (2019). Nicotinamide Phosphoribosyltransferase Aggravates Inflammation and Promotes Atherosclerosis in ApoE Knockout Mice. Acta Pharmacol. Sin 40 (9), 1184–1192. 10.1038/s41401-018-0207-3 30833708 PMC6786310

[B19] Leng B. Zhang Y. Liu X. Zhang Z. Liu Y. Wang H. (2019). Astragaloside IV Suppresses High Glucose-Induced NLRP3 Inflammasome Activation by Inhibiting TLR4/NF-Κb and CaSR. Mediators Inflamm. 2019, 1–16. 10.1155/2019/1082497 PMC6398021 30906223

[B20] Li J. Deng X. Bai T. Wang S. Jiang Q. Xu K. (2020). Resolvin D1 Mitigates Non-alcoholic Steatohepatitis by Suppressing the TLR4-MyD88-Mediated NF-Κb and MAPK Pathways and Activating the Nrf2 Pathway in Mice. Int. Immunopharmacol 88, 106961. 10.1016/j.intimp.2020.106961 33182038

[B21] Liu Z. H. Liu H. B. Wang J. (2018). Astragaloside IV Protects against the Pathological Cardiac Hypertrophy in Mice. Biomed. Pharmacother. 97, 1468–1478. 10.1016/j.biopha.2017.09.092 29793309

[B22] Ma X. Zhang K. Li H. Han S. Ma Z. Tu P. (2013). Extracts from Astragalus Membranaceus Limit Myocardial Cell Death and Improve Cardiac Function in a Rat Model of Myocardial Ischemia. J. Ethnopharmacol 149 (3), 720–728. 10.1016/j.jep.2013.07.036 23968862

[B23] Maguire E. M. Pearce S. W. A. Xiao Q. (2019). Foam Cell Formation: A New Target for Fighting Atherosclerosis and Cardiovascular Disease. Vascul Pharmacol. 112, 54–71. 10.1016/j.vph.2018.08.002 30115528

[B24] Meiqian Z. Leying Z. Chang C. (2018). Astragaloside IV Inhibits Cigarette Smoke-Induced Pulmonary Inflammation in Mice. Inflammation 41 (5), 1671–1680. 10.1007/s10753-018-0811-x 29959623

[B25] Min Li M. Li H. Fang F. Deng X. Ma S. (2017). Astragaloside IV Attenuates Cognitive Impairments Induced by Transient Cerebral Ischemia and Reperfusion in Mice via Anti-inflammatory Mechanisms. Neurosci. Lett. 639, 114–119. 10.1016/j.neulet.2016.12.046 28011393

[B26] Moore K. J. Sheedy F. J. Fisher E. A. (2013). Macrophages in Atherosclerosis: a Dynamic Balance. Nat. Rev. Immunol. 13 (10), 709–721. 10.1038/nri3520 23995626 PMC4357520

[B27] Mourikis P. Zako S. Dannenberg L. Nia A. M. Heinen Y. Busch L. (2020). Lipid Lowering Therapy in Cardiovascular Disease: From Myth to Molecular Reality. Pharmacol. Ther. 213, 107592. 10.1016/j.pharmthera.2020.107592 32492513

[B28] Nanding Wang N. Zhang X. Ma Z. Niu J. Ma S. Wenjie W. (2020). Combination of Tanshinone IIA and Astragaloside IV Attenuate Atherosclerotic Plaque Vulnerability in ApoE(-/-) Mice by Activating PI3K/AKT Signaling and Suppressing TRL4/NF-Κb Signaling. Biomed. Pharmacother. 123, 109729. 10.1016/j.biopha.2019.109729 31887543

[B29] Newman C. B. Preiss D. Tobert J. A. Jacobson T. A. Page R. L. 2nd Goldstein L. B. (2019). Statin Safety and Associated Adverse Events: A Scientific Statement from the American Heart Association. Arterioscler Thromb. Vasc. Biol. 39 (2), e38–e81. 10.1161/atv.0000000000000073 30580575

[B30] Niedzielski M. Broncel M. Gorzelak-Pabiś P. Woźniak E. (2020). New Possible Pharmacological Targets for Statins and Ezetimibe. Biomed. Pharmacother. 129, 110388. 10.1016/j.biopha.2020.110388 32559626

[B31] Okuyama H. Langsjoen P. H. Hamazaki T. Ogushi Y. Hama R. Kobayashi T. (2015). Statins Stimulate Atherosclerosis and Heart Failure: Pharmacological Mechanisms. Expert Rev. Clin. Pharmacol. 8 (2), 189–199. 10.1586/17512433.2015.1011125 25655639

[B32] Pedro-Botet J. Climent E. Benaiges D. (2020). Atherosclerosis and Inflammation. New Therapeutic Approaches. Med. Clin. (Barc) 155 (6), 256–262. 10.1016/j.medcli.2020.04.024 32571617

[B33] Purnak T. Efe C. Beyazit Y. Ozaslan E. Astan R. Milanloglu A. (2011). Recent Insights into the Relationship between Inflammatory Liver Diseases and Atherosclerosis. J. Investig. Med. 59 (6), 904–911. 10.2310/JIM.0b013e318217f3a0 21441825

[B34] Qin H. Liu P. Lin S. (2015). Effects of Astragaloside IV on the SDF-1/CXCR4 Expression in Atherosclerosis of apoE−/−Mice Induced by Hyperlipaemia. Evidence-Based Complement. Altern. Med. 2015, 1–8. 10.1155/2015/385154 PMC4449906 26074989

[B35] Quinn M. T. Parthasarathy S. Fong L. G. Steinberg D. (1987). Oxidatively Modified Low Density Lipoproteins: a Potential Role in Recruitment and Retention of Monocyte/macrophages during Atherogenesis. Proc. Natl. Acad. Sci. U S A. 84 (9), 2995–2998. 10.1073/pnas.84.9.2995 3472245 PMC304787

[B36] Roth G. A. Johnson C. Abajobir A. Abd-Allah F. Abera S. F. Abyu G. (2017). Global, Regional, and National Burden of Cardiovascular Diseases for 10 Causes, 1990 to 2015. J. Am. Coll. Cardiol. 70 (1), 1–25. 10.1016/j.jacc.2017.04.052 28527533 PMC5491406

[B37] Šimić I. Reiner Ž. (2015). Adverse Effects of Statins - Myths and Reality. Curr. Pharm. Des. 21 (9), 1220–1226. 10.2174/1381612820666141013134447 25312733

[B38] Söderberg C. Stål P. Askling J. Glaumann H. Lindberg G. Marmur J. (2010). Decreased Survival of Subjects with Elevated Liver Function Tests during a 28-year Follow-Up. Hepatology 51 (2), 595–602. 10.1002/hep.23314 20014114

[B39] Stancu C. S. Toma L. Sima A. V. (2012). Dual Role of Lipoproteins in Endothelial Cell Dysfunction in Atherosclerosis. Cell Tissue Res 349 (2), 433–446. 10.1007/s00441-012-1437-1 22592627

[B40] Sun C. Zeng G. Wang T. Ren H. An H. Lian C. (2021). Astragaloside IV Ameliorates Myocardial Infarction Induced Apoptosis and Restores Cardiac Function. Front Cel Dev Biol 9, 671255. 10.3389/fcell.2021.671255 PMC8358605 34395418

[B41] Tabas I. García-Cardeña G. Owens G. K. (2015). Recent Insights into the Cellular Biology of Atherosclerosis. J. Cel Biol 209 (1), 13–22. 10.1083/jcb.201412052 PMC4395483 25869663

[B42] Taleb S. (2016). Inflammation in Atherosclerosis. Arch. Cardiovasc. Dis. 109 (12), 708–715. 10.1016/j.acvd.2016.04.002 27595467

[B43] Tan Y. Q. Chen H. W. Li J. (2020). Astragaloside IV: An Effective Drug for the Treatment of Cardiovascular Diseases. Drug Des. Devel Ther. 14, 3731–3746. 10.2147/dddt.s272355 PMC7507407 32982178

[B44] Targher G. Bertolini L. Padovani R. Rodella S. Zoppini G. Zenari L. (2006). Relations between Carotid Artery wall Thickness and Liver Histology in Subjects with Nonalcoholic Fatty Liver Disease. Diabetes care 29 (6), 1325–1330. 10.2337/dc06-0135 16732016

[B45] Targher G. Marra F. Marchesini G. (2008). Increased Risk of Cardiovascular Disease in Non-alcoholic Fatty Liver Disease: Causal Effect or Epiphenomenon?. Diabetologia 51 (11), 1947–1953. 10.1007/s00125-008-1135-4 18762907

[B46] Targher G. Bertolini L. Padovani R. Rodella S. Zoppini G. Pichiri I. (2010). Prevalence of Non-alcoholic Fatty Liver Disease and its Association with Cardiovascular Disease in Patients with Type 1 Diabetes. J. Hepatol. 53 (4), 713–718. 10.1016/j.jhep.2010.04.030 20619918

[B47] Targher G. Day C. P. Bonora E. (2010). Risk of Cardiovascular Disease in Patients with Nonalcoholic Fatty Liver Disease. N. Engl. J. Med. 363 (14), 1341–1350. 10.1056/NEJMra0912063 20879883

[B48] Villanova N. Moscatiello S. Ramilli S. Bugianesi E. Magalotti D. Vanni E. (2005). Endothelial Dysfunction and Cardiovascular Risk Profile in Nonalcoholic Fatty Liver Disease. Hepatology 42 (2), 473–480. 10.1002/hep.20781 15981216

[B49] Wang S. Zhou H. Feng T. Wu R. Sun X. Guan N. (2014). β-Glucan Attenuates Inflammatory Responses in Oxidized LDL-Induced THP-1 Cells via the P38 MAPK Pathway. Nutr. Metab. Cardiovasc. Dis. 24 (3), 248–255. 10.1016/j.numecd.2013.09.019 24418375

[B50] Wei R. Liu H. Chen R. Sheng Y. Liu T. (2019). Astragaloside IV Combating Liver Cirrhosis through the PI3K/Akt/mTOR Signaling Pathway. Exp. Ther. Med. 17 (1), 393–397. 10.3892/etm.2018.6966 30651810 PMC6307369

[B51] Wu Y. Wang F. Fan L. Zhang W. Wang T. Du Y. (2018). Baicalin Alleviates Atherosclerosis by Relieving Oxidative Stress and Inflammatory Responses via Inactivating the NF-Κb and P38 MAPK Signaling Pathways. Biomed. Pharmacother. 97, 1673–1679. 10.1016/j.biopha.2017.12.024 29793330

[B52] Xin Y. Li G. Liu H. Ai D. (2015). AS-IV Protects against Kidney IRI through Inhibition of NF-Κb Activity and PUMA Upregulation. Int. J. Clin. Exp. Med. 8 (10), 18293–18301. 26770431 PMC4694331

[B53] Xiong-Zhi Li X. Z. Ding Y. Z. Wu H. F. Bian Z. P. Xu J. D. Gu C. R. (2017). Astragaloside IV Prevents Cardiac Remodeling in the Apolipoprotein E-Deficient Mice by Regulating Cardiac Homeostasis and Oxidative Stress. Cell Physiol Biochem 44 (6), 2422–2438. 10.1159/000486166 29268252

[B54] Xu W. Shao X. Tian L. Gu L. Zhang M. Wang Q. (2014). Astragaloside IV Ameliorates Renal Fibrosis via the Inhibition of Mitogen-Activated Protein Kinases and Antiapoptosis *In Vivo* and *In Vitro* . J. Pharmacol. Exp. Ther. 350 (3), 552–562. 10.1124/jpet.114.214205 24951279

[B55] Xu X. Gao W. Cheng S. Yin D. Li F. Wu Y. (2017). Anti-inflammatory and Immunomodulatory Mechanisms of Atorvastatin in a Murine Model of Traumatic Brain Injury. J. Neuroinflammation 14 (1), 167. 10.1186/s12974-017-0934-2 28835272 PMC5569493

[B56] Yan W. Xu Y. Yuan Y. Tian L. Wang Q. Xie Y. (2017). Renoprotective Mechanisms of Astragaloside IV in Cisplatin-Induced Acute Kidney Injury. Free Radic. Res. 51 (7-8), 669–683. 10.1080/10715762.2017.1361532 28750561

[B57] Yang L. Han X. Yuan J. Xing F. Hu Z. Huang F. (2020). Early Astragaloside IV Administration Attenuates Experimental Autoimmune Encephalomyelitis in Mice by Suppressing the Maturation and Function of Dendritic Cells. Life Sci. 249, 117448. 10.1016/j.lfs.2020.117448 32087232

[B58] Yang X. Wang F. (2019). The Effect of Astragaloside IV on JAK2-STAT6 Signalling Pathway in Mouse Model of Ovalbumin-Induced Asthma. J. Anim. Physiol. Anim. Nutr. (Berl) 103 (5), 1578–1584. 10.1111/jpn.13114 31148265

[B59] Yiru Wang Y. Jia Q. Zhang Y. Wei J. Liu P. (2020). Taoren Honghua Drug Attenuates Atherosclerosis and Plays an Anti-inflammatory Role in ApoE Knock-Out Mice and RAW264.7 Cells. Front. Pharmacol. 11, 1070. 10.3389/fphar.2020.01070 32765273 PMC7379336

[B60] Yu S. Y. Ouyang H. T. Yang J. Y. Huang X. L. Yang T. Duan J. P. (2007). Subchronic Toxicity Studies of Radix Astragali Extract in Rats and Dogs. J. Ethnopharmacol 110 (2), 352–355. 10.1016/j.jep.2006.09.024 17052876

[B61] Yun K. E. Shin C. Y. Yoon Y. S. Park H. S. (2009). Elevated Alanine Aminotransferase Levels Predict Mortality from Cardiovascular Disease and Diabetes in Koreans. Atherosclerosis 205 (2), 533–537. 10.1016/j.atherosclerosis.2008.12.012 19159884

[B62] Zhang W. J. Frei B. (2015). Astragaloside IV Inhibits NF- κ B Activation and Inflammatory Gene Expression in LPS-Treated Mice. Mediators Inflamm. 2015, 274314. 10.1155/2015/274314 25960613 PMC4415625

[B63] Zhang L. Yang Y. Wang Y. Gao X. (2011). Astragalus Membranaceus Extract Promotes Neovascularisation by VEGF Pathway in Rat Model of Ischemic Injury. Pharmazie 66 (2), 144–150. 21434579

[B64] Zhang Y. Zhang Y. Jin X. F. Zhou X. H. Dong X. H. Yu W. T. (2019). The Role of Astragaloside IV against Cerebral Ischemia/Reperfusion Injury: Suppression of Apoptosis via Promotion of P62-LC3-Autophagy. Molecules 24 (9). 10.3390/molecules24091838 PMC6539971 31086091

[B65] Zhang J. Wu C. Gao L. Du G. Qin X. (2020). Astragaloside IV Derived from Astragalus Membranaceus: A Research Review on the Pharmacological Effects. Adv. Pharmacol. 87, 89–112. 10.1016/bs.apha.2019.08.002 32089240

[B66] Zhong L. Simard M. J. Huot J. (2018). Endothelial microRNAs Regulating the NF-Κb Pathway and Cell Adhesion Molecules during Inflammation. FASEB J. 32 (8), 4070–4084. 10.1096/fj.201701536R 29565737

