# Supplementary figures and images for: Astragaloside IV Relieves Atherosclerosis and Hepatic Steatosis via MAPK/NF-κB Signaling Pathway in LDLR−/− Mice
Source: Front Pharmacol. 2022 Feb 21;13:828161. doi: 10.3389/fphar.2022.828161 (PMC8899310; doi:10.3389/fphar.2022.828161)

Supplementary Material

# Animal welfare review material


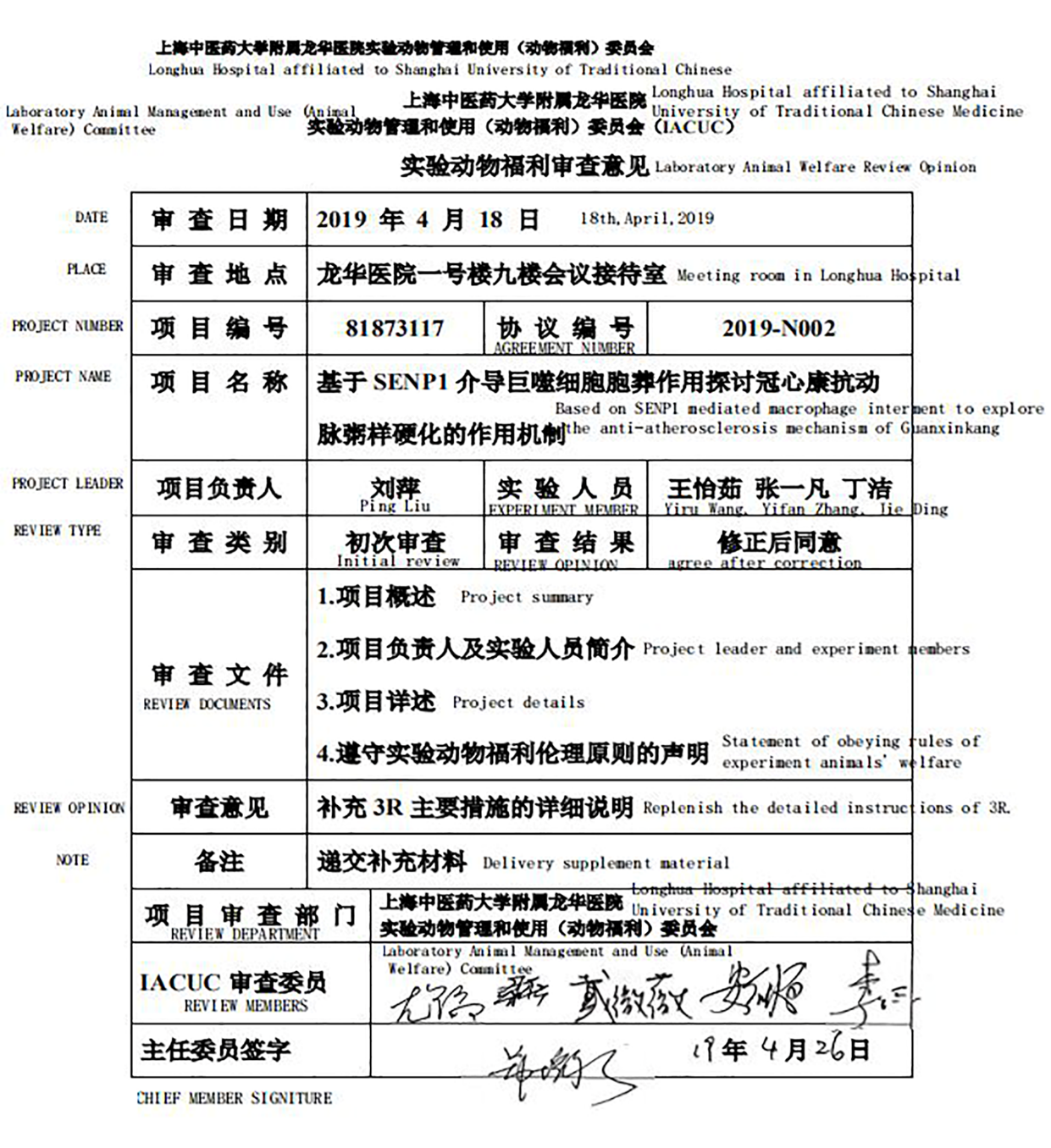

Supplement: Supplementary file 1 [file Table1.DOCX]
